# What Does Bacteria Have to Do with Cancer? The Influence of the Body’s Microbiota on Cancer in Cats and Dogs

**DOI:** 10.3390/ijms27115005

**Published:** 2026-06-01

**Authors:** Patrycja Kasperska, Iga Horodyska, Julia Mateja, Aleksandra Sobierajewicz, Marta Miszczak, Karolina Bierowiec, Joanna Bubak

**Affiliations:** 1 EZA Student Science Club, Department of Epizootiology and Clinic of Birds and Exotic Animals, Faculty of Veterinary Medicine, Wrocław University of Environmental and Life Sciences, Grunwaldzki Sq. 45, 50-366 Wrocław, Poland; 120498@student.upwr.edu.pl (I.H.); 120501@student.upwr.edu.pl (J.M.); 120450@student.upwr.edu.pl (A.S.); 2 Department of Epizootiology and Clinic of Birds and Exotic Animals, Division of Infectious Diseases and Veterinary Administration, Faculty of Veterinary Medicine, Wrocław University of Environmental and Life Sciences, Grunwaldzki Sq. 45, 50-366 Wrocław, Poland; marta.miszczak@upwr.edu.pl (M.M.); karolina.bierowiec@upwr.edu.pl (K.B.); 3 Department of Pathology, Division of Pathomorphology and Veterinary Forensics, Faculty of Veterinary Medicine, Wrocław University of Environmental and Life Sciences, 31 Norwida St., 50-375 Wrocław, Poland; joanna.bubak@upwr.edu.pl

**Keywords:** microbiota in cancer, bacterial therapy, oncolytic bacteria

## Abstract

The body’s microbiota plays a fundamental role in maintaining homeostasis and influences immune function, metabolism, and tissue integrity. A growing body of research suggests that fluctuations in the composition and abundance of individual microbiota populations may influence cancer development and the effectiveness of therapy. The condition of microbiota dysbiosis has been demonstrated to induce chronic inflammation, immune system dysregulation, and, most significantly, modulation of molecular pathways that promote tumorigenesis. The efficacy and toxicity of cancer treatment can be influenced by the composition of the microbiota. Bacteria can modify the effectiveness and toxicity of chemotherapy and immunotherapy by affecting drug metabolism and the body’s immune response. In contrast, the development of anticancer therapies that utilize bacteria is gaining increasing interest. This alternative to conventional treatment utilizes the natural ability of certain bacterial species to selectively colonize hypoxic and necrotic environments. The exploration of natural and genetically modified bacteria as vectors for the delivery of cytotoxins, immunomodulators, or therapeutic genes in the combat of cancer is a current area of research. In addition, their capacity to stimulate an antitumor immune response is also exploited. Preclinical investigations in animals have demonstrated the efficacy of this therapeutic approach, underscoring the promise of bacterial therapies as either an adjunct to conventional treatment or as a standalone strategy for combating cancer. This article synthesizes the current knowledge regarding the role of microbiota in carcinogenesis in animals and discusses recent developments in the field of bacterial therapies. The text also addresses the challenges, safety considerations, and future perspectives associated with translating microbiota-targeted and bacterial therapies into veterinary and comparative oncology.

## 1. Introduction

The status of companion animals has evolved significantly, with pets now predominantly viewed as integral members of the family who provide vital emotional and social support, effectively reducing loneliness and fostering human connections [1]. This profound connection has motivated owners to prioritize optimal welfare and cutting-edge healthcare, while concurrently serving as a pivotal catalyst for research into human well-being.

As the role of companion animals in human life continues to expand and their lifespan increases, growing attention must also be paid to the health challenges they face. While the benefits of human–animal interactions are well documented, the increasing longevity of companion animals has led to a higher prevalence of chronic and age-related diseases, which pose significant challenges for veterinary care and animal welfare. Research has demonstrated that approximately one in four dogs and one in five cats are predisposed to developing cancer during their lifetime [2,3,4]. In many cases, the available treatment options remain limited, highlighting the need for further research aimed at improving cancer diagnostics and therapeutic approaches. Cancer is a leading cause of mortality in dogs and cats, and its clinical significance is steadily increasing with the increasing life expectancy of companion animals and advances in veterinary diagnostics [5,6]. It has been estimated that the risk of developing cancer increases considerably in geriatric animals, particularly those over 10 years of age [7]. The clinical presentation of these conditions exhibits significant variability, a factor that complicates the process of diagnosis and treatment. The most prevalent types of cancer diagnosed in dogs include cutaneous and subcutaneous lesions (including mastocytoma and melanoma), mammary gland tumors, lymphomas, and tumors of solid organs such as the spleen and liver. In female dogs, mammary gland tumors represent one of the most prevalent forms of cancer [6,8,9]. In cats, lymphomas, mammary gland tumors, and soft tissue sarcomas comprise a substantial category, frequently exhibiting a more aggressive course compared to dogs [6,10]. Cancers of the oral cavity and perianal glands are also relatively common in both species [6]. The etiopathogenesis of cancer in companion animals is multifactorial, involving both genetic and environmental factors [6]. Breed predisposition plays a significant role, particularly in purebred dogs, which exhibit an elevated risk of certain types of cancer [7]. Hormonal status has also been identified as a significant risk factor. Environmental factors, including exposure to UV radiation, dietary influences, persistent inflammation, and oxidative stress, have been identified as significant contributors to the development and progression of cancer [5,6]. In recent years, there has been a notable advancement in the field of veterinary oncology, particularly in the development of diagnostic and prognostic methods. These methods include the utilization of cell proliferation indices, which have demonstrated a substantial correlation with patient survival across various cancer types [11]. Despite the advancements in diagnostic methods, the early detection of neoplastic lesions remains a clinical challenge, underscoring the significance of screening programs and the identification of risk factors. An increasing body of scientific evidence suggests that bacteria play a substantial role in oncogenic processes [11].

Disruptions in the composition of the microbiome have been associated with the development of numerous diseases in both animals and humans [12]. Advancements in microbiome research have enabled a better understanding of its impact on animal health, physiology, and behavior. In humans, shifts in the composition of the gut microbiota have been demonstrated to be strongly associated with the development of inflammatory bowel diseases (IBD), including Crohn’s disease and ulcerative colitis, resulting from abnormal interactions between microorganisms and the immune system [13,14]. Furthermore, dysbiosis plays a significant role in the pathogenesis of irritable bowel syndrome and celiac disease, affecting gastrointestinal function and inflammatory processes [15,16]. The impact of changes in the microbiome on allergic diseases such as asthma has been further investigated, and it has been demonstrated that these changes have a significant impact on immune regulation. Additionally, a link has been established between the microbiome and carcinogenesis, particularly colorectal cancer, where it has been shown that the microbiome can promote chronic inflammation and tumor development [17,18].

In animals, particularly companion animals such as dogs and cats, dysbiosis has been demonstrated to result in digestive disorders and chronic inflammatory enteropathies resembling human IBD [19]. Moreover, it increases susceptibility to infections and systemic diseases by compromising intestinal barrier function and the immune response [19]. Research conducted in animal models has demonstrated a correlation between this condition and obesity, diabetes, and allergic diseases. This finding highlights the notion that the microbiome is a common factor in the pathogenesis of many diseases across species [18].

The complex interactions between the host, its microbiome, and health status are the focal point of an increasing number of studies and scientific reports. The characterization of microbial communities has the potential to establish a foundation for the development of novel and more effective strategies for disease prevention and health promotion in companion animals. Understanding the role of bacteria in the context of cancer in companion animals is of considerable importance not only for elucidating disease mechanisms but also for the development of diagnostic and therapeutic tools in veterinary oncology.

Notably, the microbiota serves not only as a diagnostic indicator of cancer development, but also as a potential therapeutic agent [20,21]. The targeted modulation of tumor microbiota and bacteriotherapy represents a promising, although still intensively researched, direction for the development of modern oncological treatment strategies [21].

This review aims to provide a concise summary of the current knowledge regarding the role of bacteria and the microbiome in cancer development in cats and dogs, along with their potential application in diagnostic and therapeutic strategies in veterinary oncology.

## 2. Bacteria as a Risk Factor for Cancer

In dogs and cats, the microbiota, similarly to humans, constitutes a complex ecosystem comprising microorganisms that fulfill pivotal functions in metabolism, immune system function, and maintaining body homeostasis [22]. Each anatomical region contains a distinct composition of bacterial genera and species [23,24]. In healthy animals, the gut microbiota is predominantly comprised of the phyla *Firmicutes*, *Bacteroidetes*, *Proteobacteria*, *Fusobacteria*, and *Actinobacteria* [25]. The skin is colonized by 85% of bacteria from the phyla *Proteobacteria*, *Bacteroidota*, and *Actinobacteria* [23,26]. The microbiota of the reproductive tract exhibits significant differences between female and male dogs. In female dogs, the predominant microbiota include the phylum *Fusobacteriota* and the genera *Ralstonia*, *Hydrotalea*, and *Mycoplasma* [23,27]. In contrast, male dogs predominantly harbor the phyla *Proteobacteria*, *Firmicutes* and *Actinobacteria* [23,27]. It is evident that each anatomical region is characterized by a distinct microbiome composition, which performs a specific function. In healthy animals, bacteria exist in a eubiotic state and perform various functions, ranging from forming a protective barrier, aiding digestion, synthesizing health-promoting metabolites, and influencing the development and function of the immune system [22,28].

In the context of cancer in dogs and cats, an increasing number of studies suggest that alterations in microbiota composition (dysbiosis) may accompany or potentially contribute to tumor development [20]. Several reports have demonstrated an association between microbiota alterations, particularly within the intestinal microbiota, and the occurrence of neoplastic diseases [29,30]. These alterations have been linked to disruptions in microbial metabolite production and the activation of signaling pathways that play a role in inflammation and carcinogenesis [31]. However, the majority of currently available veterinary studies are observational in nature, and direct causal relationships between microbiota dysbiosis and tumorigenesis remain insufficiently established.

### 2.1. Mechanisms of Bacteria-Induced Carcinogenesis

Cancer is one of the leading causes of morbidity and mortality in humans and animals. Its etiology is the result of complex interactions between genetic, environmental, and biological factors, including microbial infections [32]. In recent years, increasing attention has been directed toward the role of bacteria as potential risk factors for cancer, particularly in the context of chronic infections and disruptions of the microbiota composition [33].

**Figure 1:**
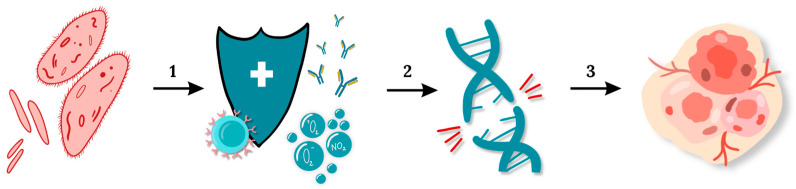
**Bacteria-induced carcinogenesis:** 1—Bacteria and their metabolites, through the induction of chronic inflammation, modulate the host immune response, 2—This process leads to prolonged activation of immune cells and increased production of reactive oxygen and nitrogen species, which can cause DNA damage and impair DNA repair mechanisms, 3—DNA damage combined with chronic inflammation promotes neoplastic cell transformation [21,33].

It has been hypothesized that disturbances in the microbiota composition may influence the development of cancer through several interconnected pathways. Dysbiosis has been demonstrated to contribute to the induction of chronic inflammation, the production of genotoxic metabolites, modulation of the host immune response, and effects on the tumor microenvironment (TME). Collectively, these factors could create conditions favorable for tumor initiation and progression (Figure 1) [32,34,35]. An important role in diseases in dogs and cats is attributed to metabolites of the intestinal microbiota. These metabolites include short-chain fatty acids (SCFA), secondary bile acids, branched-chain fatty acids (BCFA), indoles, biogenic amines, phenols, p-cresol and hydrogen sulfide. These compounds have the potential to influence inflammatory responses, the integrity of the intestinal barrier and cellular proliferation [22]. A significant number of these metabolites exhibit similarities with those observed in human colon cancer models. However, disparities are more likely related to variations in proportions and concentrations, as well as the predominant metabolic pathways [36,37]. The overrepresentation of potentially pathogenic or pro-inflammatory bacteria (including *Escherichia*, *Shigella*, *Clostridium*, and *Bacteroides* in excess) has also been associated with toxin production and changes in the TME, such as altered pH, hypoxia, and local immunosuppression, which may favor cancer progression [38,39].

The clinical observations are further supported by several proposed molecular mechanisms that potentially link microbiota dysbiosis with cancer initiation and progression [40,41,42]. These factors are presented in Table 1.

One proposed mechanism involves chronic inflammation resulting from an imbalance between commensal and proinflammatory bacterial taxa [35,43]. The increased abundance of *Proteobacteria*, along with the reduction in beneficial commensals, has been associated with the activation of inflammatory pathways, including Nuclear factor-κB (NF-κB) signaling and increased production of cytokines such as IL-6 and TNF-α [44,45,46]. Persistent inflammatory signaling may contribute to increased cell proliferation, genomic instability, and the formation of a microenvironment that is permissive for neoplastic transformation [34,47].

Another proposed mechanism involves impaired intestinal barrier integrity [25]. A decrease in the abundance of SCFA-producing bacteria may result in a reduction in epithelial energy supply and a weakening of tight junction function, potentially facilitating the translocation of bacterial components such as lipopolysaccharide (LPS) [22,28]. The depletion of SCFAs, particularly butyrate, has also been demonstrated to reduce anti-inflammatory and antiproliferative signaling [22]. Furthermore, the hypothesis that dysregulated bile acid metabolism increases concentrations of secondary bile acids with mutagenic and pro-oxidative properties has been advanced, largely based on comparative animal and human studies [20].

Altered microbial tryptophan metabolism may also affect aryl hydrocarbon receptor (AhR) signaling, potentially impairing immune tolerance and facilitating immune evasion by neoplastic cells [34,48]. Beyond local intestinal effects, microbiota-derived metabolites and cytokines may exert systemic influences [20,49]. The presence of inflammatory mediators and microbial metabolites in the circulation has the potential to modify the TME in distant tissues by promoting angiogenesis, immune escape, and tumor progression, even in organs not directly colonized by the dysbiotic microbiota [20,50].

**Table 1:** Molecular mechanisms linking microbiota dysbiosis to carcinogenesis in dogs and cats.

Molecular Mechanism	Microbiota Alteration	Key Mediators	Biological Effect	Role in Carcinogenesis	References
Chronic inflammation	↑ *Proteobacteria*; ↓ commensal bacteria	IL-6, TNF-α, NF-κB	Sustained immune activation	Promotion of proliferation and mutagenesis	[45,51,52,53]
Loss of intestinal barrier integrity	↓ SCFA-producing bacteria	Zonulin, LPS	Increased microbial translocation	Persistent stimulation of gut-associated lymphoid tissue (GALT); lymphoma risk	[45,54,55]
SCFA depletion	↓ *Faecalibacterium*, *Blautia*	Butyrate, propionate	Reduced anti-inflammatory and antiproliferative signaling	Facilitation of neoplastic transformation	[34,35,43,56]
Tryptophan metabolism dysregulation	Gut dysbiosis	Indoles, AhR pathway	Impaired immune tolerance	Immune escape of neoplastic cells	[48,57,58,59]
Altered bile acid metabolism	Microbial composition shifts	Deoxycholic acid	Oxidative stress, DNA damage	Mutagenic effects promoting cancer	[58,60,61]
Impaired immune surveillance	Microbiota imbalance	Treg/Th17 imbalance	Reduced elimination of atypical cells	Survival and expansion of malignant clones	[51,62,63]
Systemic effects of microbial metabolites	Gut dysbiosis	Circulating cytokines and metabolites	Altered TME in distant tissues	Influence on extraintestinal cancers	[55,64,65]

### 2.2. Helicobacter and Gastric Carcinogenesis

The gut microbiota of animals also plays a significant role in cancer pathogenesis, exhibiting immunomodulatory and metabolic functions under physiological conditions [66]. Disruptions in microbiota composition, referred to as dysbiosis, have been shown to lead to increased intestinal barrier permeability, bacterial translocation, and an enhanced inflammatory response, thereby promoting tumorigenic processes [21]. In companion animals, particularly dogs and cats, significant differences in gut microbiota composition have been observed between healthy individuals and those with gastrointestinal cancers, including intestinal lymphomas [52]. Translational studies have indicated that dogs with intestinal lymphoma exhibit reduced microbial diversity and a predominance of potentially pro-inflammatory bacteria (*Escherichia coli*, *Clostridium perfringens*), which may promote chronic intestinal mucosal inflammation and tumorigenic transformation [21]. Corresponding associations have been documented in studies of colorectal cancer in humans, underscoring the significance of cross-species comparisons in the realm of microbiome research [32].

One of the most thoroughly characterized examples of bacterial carcinogenesis in humans is *Helicobacter pylori* (*H. pylori*), a spiral-shaped, Gram-negative bacterium capable of colonizing the acidic gastric environment due to its urease activity [67]. Approximately 50% of the global human population is infected with *H. pylori*, a gastric bacterium. This infection has been definitively linked to the development of gastric cancer and Mucosa-Associated Lymphoid Tissue-type (MALT-type) lymphoma [68]. The International Agency for Research on Cancer (IARC) has classified *H. pylori* as a Group 1 carcinogen, indicating a confirmed causal relationship with cancer development [69]. The mechanisms of *H. pylori*-induced carcinogenesis include both chronic gastric mucosal inflammation and the direct effects of bacterial virulence factors on host cells [67]. The cytotoxin-associated gene A protein (CagA protein) disrupts cellular signaling, promotes cell proliferation, and inhibits apoptosis. The Vacuolating cytotoxin A (VacA toxin) induces cellular vacuolization and modulates the immune response [70]. Prolonged infection results in a series of pathological changes, including atrophic gastritis, intestinal metaplasia, dysplasia, and ultimately carcinoma [71]. In the veterinary context, *H. pylori* is rarely detected in dogs and cats; however, other species of the genus *Helicobacter*, referred to as non-*Helicobacter pylori Helicobacters* (NHPH), such as *H. felis*, *H. heilmannii*, *H. bizzozeronii*, and *H. salomonis*, are much more commonly identified in these species [72]. These bacteria colonize the gastric mucosa in both healthy animals and individuals exhibiting gastrointestinal symptoms, which complicates the clear determination of their pathogenicity [73]. A further limitation is the current lack of specific diagnostic markers for these bacteria. The clinical significance of infection is predominantly determined by the severity of histopathological alterations and the host inflammatory response (including IL-1β and TNF-α) [72]. However, there are reports of the presence of *Helicobacter* spp. in the gastric tissues of dogs and cats with chronic gastritis and MALT-type lymphoma, suggesting a potential role for these bacteria in tumorigenic processes through mechanisms analogous to those observed in humans [74]. It has been demonstrated that the presence of *Helicobacter* spp. can induce an inflammatory response that may contribute to alterations in cellular homeostasis, an escalation in oxidative stress, and genomic instability [72]. Understanding the role of bacteria and the microbiota as risk factors for cancer in animals has important clinical implications, as it may enable the identification of predictive biomarkers, the development of preventive strategies, and the modulation of the microbiota through diet, probiotics, or therapeutic interventions [21]. Although many mechanisms have been more thoroughly described in human medicine, an increasing number of veterinary studies indicate that bacterial carcinogenesis represents a significant and shared aspect of tumor biology across various mammalian species [33].

### 2.3. Gut Dysbiosis in Gastrointestinal Cancers

The association between altered microbiota composition and cancer development in animals has been most extensively investigated in gastrointestinal neoplasms, particularly intestinal lymphomas. However, growing evidence suggests that dysbiosis may also exert systemic immunometabolic effects potentially relevant to dysbiosis in extraintestinal cancers [34,56,75].

In cats, low-grade intestinal T-cell lymphoma (LGITL) is considered a well-described veterinary model that links chronic intestinal inflammation, dysbiosis, and neoplastic disease [43]. Cats with intestinal lymphoma have been reported to exhibit reduced microbial diversity, decreased abundance of SCFA-producing bacteria such as *Faecalibacterium*, and an increased representation of *Proteobacteria* [51,76]. A similar microbiota alteration has also been observed in cats with IBD, supporting the hypothesis of a possible pathological continuum between chronic enteropathy and lymphoma development [43]. Beyond these compositional parallels, recent metabolomic investigations have revealed that both cats with chronic inflammatory enteropathy (CIE) and LGITL exhibit significantly diminished concentrations of microbiota-derived indole metabolites of tryptophan, including indolepropionate, indoleacrylate, indolealdehyde, and indolelactate, in comparison with healthy controls [57]. These metabolites are involved in maintaining intestinal epithelial integrity and regulating mucosal immune responses through AhR-dependent signaling pathways, suggesting that their depletion may contribute to persistent inflammation and impaired immune homeostasis [77]. A notable finding was the significantly lower indolelactate concentrations observed in cats with LGITL compared to those with CIE. This observation suggests a progressive disruption of microbiota–host metabolic interactions during the neoplastic transformation process [57]. The findings, considered collectively, provide substantiating evidence for a hypothesis that chronic immune stimulation associated with dysbiosis may promote the gradual selection and expansion of neoplastic T-cell clones. However, prospective longitudinal studies are still required to establish direct causality [57,76].

Long-term clinical observations suggest that microbiota alterations may occur before the diagnosis of lymphoma in some cats [43]. Retrospective analyses in dogs have similarly indicated that microbiota abnormalities can sometimes be detected months or years before lymphoma diagnosis. However, these findings do not establish causality and require confirmation in prospective longitudinal studies [51].

The following table (Table 2) synthesizes data from clinical studies and case reports describing associations between microbiota alterations and the development or clinical course of cancer in dogs and cats.

In dogs, the severity of microbiota alterations has been reported to correlate with tumor aggressiveness [51]. Dogs with high-grade intestinal lymphoma or rapidly progressing gastrointestinal tumors frequently exhibit more pronounced dysbiosis compared to those with indolent or localized tumors [34]. These patients may also present with severe systemic manifestations, including weight loss, hypoalbuminemia, and anemia, which could partly reflect chronic inflammation and altered microbial metabolism [22,28]. Dysbiosis has also been documented in dogs with extraintestinal malignancies, including multicentric lymphoma [20]. Despite the absence of primary intestinal lesions, affected dogs may exhibit significant alterations in gut microbiota composition that correlate with systemic inflammatory markers [20]. These findings support the hypothesis that the gut microbiota may influence systemic immunometabolic pathways, which could be relevant to tumor biology at distant sites [20].

### 2.4. Extraintestinal Cancers and Systemic Effects

A number of mechanisms have been postulated to demonstrate how intestinal dysbiosis may influence the development and progression of extraintestinal neoplasms through so-called gut-organ axes [60]. Microbiota-derived metabolites and bacterial products, including SCFAs, secondary bile acids, LPS, and tryptophan metabolites, have the capacity to enter the systemic circulation and modulate immune responses, inflammatory signaling, and epithelial homeostasis in distant tissues [81,82]. Dysbiosis-associated alterations in immune regulation may additionally affect the activity of regulatory T cells, Th17 lymphocytes, macrophages, and natural killer (NK) cells, thereby contributing to chronic systemic inflammation and changes in antitumor immunity [83]. These systemic immunometabolic effects may subsequently modify the TME by promoting angiogenesis, immune evasion, oxidative stress, and pro-inflammatory signaling pathways in organs not directly colonized by the altered microbiota [84]. The existence of analogous mechanisms has been postulated in various gut-organ communication pathways, such as the gut–mammary gland, gut–lung, and gut–liver axes, where the influence of intestinal microbiota on tumor biology may be indirect, manifested through circulating metabolites and immune mediators [84,85]. However, direct experimental evidence supporting these mechanisms in canine and feline oncology remains limited, and many of the currently proposed pathways are based primarily on findings from human and murine studies.

### 2.5. Skin Microbiome and Carcinogenesis

Beyond the gastrointestinal tract, increasing attention is being paid to the skin microbiome in the context of cutaneous cancers in animals [80]. In dogs and cats affected by cutaneous squamous cell carcinoma (SCC), alterations in the skin microbiota have been observed, specifically a reduction in microbial diversity. This phenomenon is accompanied by a relative predominance of bacteria from the genus *Staphylococcus*, suggesting a shift toward dysbiosis associated with tumor-induced changes in the cutaneous microenvironment. However, there is currently no conclusive evidence supporting its primary role in carcinogenesis [86]. Chronic skin inflammation induced by bacterial colonization is considered a factor that promotes DNA damage in keratinocytes and may contribute to the development of neoplastic lesions [32].

### 2.6. Gut Dysbiosis and Clinical Outcome During Chemotherapy

A body of research has indicated that the composition of the microbiota may have a role in determining treatment tolerance and clinical outcomes in dogs undergoing chemotherapy [20]. Dogs with less severe dysbiosis before treatment have been documented to exhibit a reduced frequency of gastrointestinal adverse effects and, in some cases, prolonged progression-free intervals [20]. A number of studies have demonstrated that alterations in the composition of the gut microbiota are associated with chemotherapy-induced gastrointestinal (GI) toxicity in dogs undergoing CHOP-based protocols (cyclophosphamide, doxorubicin, vincristine, and prednisone) [79,87]. In particular, a state of dysbiosis characterized by a decrease in the abundance of commensal, anti-inflammatory taxa such as *Faecalibacterium*, *Fusobacterium*, *Blautia*, *Turicibacter*, and *Clostridium hiranonis* has been consistently reported in dogs diagnosed with lymphoma that are undergoing chemotherapy. In contrast, an increase in potentially pro-inflammatory or opportunistic taxa, including *Escherichia coli* and *Streptococcus*, is commonly observed in states of dysbiosis and has been associated with more pronounced gastrointestinal clinical signs, including diarrhea [1].

Prospective studies in dogs receiving CHOP chemotherapy have demonstrated that treatment is associated with an increase in dysbiosis index scores, reflecting a shift away from beneficial SCFA-producing bacteria (e.g., *Faecalibacterium prausnitzii* group) and toward facultative anaerobes such as *Enterobacteriaceae*, including *E. coli*, which has been linked to intestinal inflammation and diarrhea episodes during chemotherapy cycles [87]. Similarly, a decrease in *Clostridium hiranonis* has been linked to impaired bile acid metabolism, which may contribute to altered intestinal fluid secretion and chemotherapy-induced diarrhea [87,88,89].

Clinical studies evaluating vincristine- and doxorubicin-containing CHOP protocols have also demonstrated that dogs with a more stable microbiota composition prior to treatment, characterized by a higher relative abundance of *Firmicutes* members such as *Faecalibacterium* and *Blautia*, tend to experience fewer gastrointestinal adverse events, including lower-grade diarrhea and vomiting, compared to dogs exhibiting baseline dysbiosis [87,90]. Conversely, an increased abundance of *Proteobacteria* (particularly *Enterobacteriaceae*) has been associated with higher dysbiosis index values and a greater likelihood of clinically relevant GI toxicity [87].

Although the majority of current studies have focused on compositional shifts rather than direct causal pathways, emerging evidence suggests that chemotherapy-induced alterations in microbiota may contribute to GI toxicity through the disruption of microbial metabolites (e.g., SCFA) and bile acid homeostasis. This disruption can lead to the intensification of mucosal inflammation and epithelial barrier dysfunction. However, the majority of current data derive from small prospective cohorts, and robust associations between individual bacterial taxa and specific clinical endpoints (e.g., vomiting vs. diarrhea severity during CHOP) remain incompletely defined and require further controlled validation [87,90].

These observations have led to an increased interest in microbiota-targeted therapeutic approaches, including probiotics, prebiotics, and fecal microbiota transplantation (FMT), as potential adjuncts to conventional anticancer therapy [51,65,91]. Preliminary findings suggest that such strategies may improve treatment tolerance and support overall health status; however, controlled prospective clinical trials are still required to determine their efficacy and clinical relevance in veterinary oncology [51,65].

## 3. Oncolytic Bacteria and Bacteriobots in Cancer Therapy

Oncolytic bacteria are a type of bacterium that have been either genetically modified or are naturally occurring. These bacteria have the ability to selectively colonize and destroy cancer cells while limiting their impact on healthy tissue [92,93]. These agents represent a promising class of biological anticancer agents. They are being developed as an alternative or complement to traditional cancer treatments such as chemotherapy, radiotherapy, and immunotherapy [94,95]. The selectivity of oncolytic bacteria is influenced by the characteristics of the TME, including hypoxia, low pH, necrosis, and a compromised immune response, which favor bacterial proliferation [92,93]. The most frequently studied species include anaerobic or facultatively anaerobic bacteria, which can be further genetically modified to enhance safety and therapeutic efficiency [96,97]. The mechanisms of oncolytic bacteria include direct lysis of cancer cells, competition for nutrients, production of toxins and enzymes that degrade tumor tissue, and induction of an immune response directed against the tumor [98]. A significant element of their mechanism is the capacity to stimulate the host’s innate and adaptive immune responses, which may result in a systemic effect and the elimination of metastases [93].

Oncolytic bacteria can act on tumors in several complementary ways. A considerable number of solid tumors are distinguished by a specific microenvironment, characterized by conditions that may be oxygen-poor (hypoxic) or necrotic. This creates a niche in which anaerobic or facultatively anaerobic bacteria can proliferate, while healthy, well-oxygenated tissues do not support their growth [92]. Anaerobic bacteria of the *Clostridium* genus, due to their capacity for spore formation, exhibit a notable ability to germinate exclusively within hypoxic/necrotic tumor regions, minimizing the risk of infection of normal tissues [99].

Despite the fact that oncolytic bacteria demonstrate a preference for colonizing hypoxic and necrotic tumor regions, recent advancements in engineering methodologies have led to an expansion in the applicability of these bacteria to highly vascularized and earlier-stage tumors that do not exhibit extensive necrotic cores [100]. Facultative anaerobic species such as *Salmonella typhimurium* possess the capacity to survive within both oxygenated and hypoxic tumor regions, allowing penetration beyond severely necrotic zones [101]. Furthermore, the phenomenon of tumor tropism is influenced not only by hypoxia but also by aberrant vascular permeability, impaired lymphatic drainage, immune suppression, and tumor-associated metabolite gradients. These characteristics may facilitate selective bacterial accumulation even in tumors with heterogeneous oxygenation profiles [100,102].

Recent advancements in synthetic biology have enabled the development of engineered bacterial sensors capable of responding dynamically to tumor-associated environmental signals beyond hypoxia alone. The reported systems include promoters that are responsive to oxygen tension, pH, lactate concentration, quorum density, and externally inducible stimuli [103,104]. These biosensing platforms enable the selective activation of cytotoxic payloads, immune stimulators, or bacteriolytic systems only after their precise localization within tumor tissue. Programmable circuits incorporating hypoxia-sensitive promoters, synchronized lysis systems, and quorum-sensing mechanisms have been proposed to improve tumor specificity while limiting off-target colonization and systemic inflammation [104,105,106]. These engineered sensing systems have the potential to expand the therapeutic applications of oncolytic bacteria to tumors that exhibit limited necrosis or increased vascularization, while simultaneously improving biosafety and controllability.

Some oncolytic bacteria have been shown to directly destroy tumor cells through lytic activity, the production of toxins or proteolytic enzymes, or by inducing cellular stress leading to cell death (e.g., apoptosis, autophagy) [107]. It has been demonstrated that *Clostridium novyi*-NT, *S. typhimurium*, *E. coli*, and *Listeria monocytogenes* exhibit the capacity to directly destroy tumor cells through mechanisms that ultimately result in apoptosis. *Clostridium novyi*-NT produces toxins that cause damage to cellular structures under conditions of hypoxia, triggering a cellular stress response that leads to cell death, including apoptosis. *Salmonella typhimurium* has been observed to induce oxidative stress and disrupt cellular functions, thereby activating apoptotic pathways. Similarly, genetically modified strains of *E. coli* produce toxins that interfere with the cell cycle and compromise cellular integrity, leading to the initiation of apoptosis. In turn, *L. monocytogenes*, by invading tumor cells and disturbing their homeostasis, induces stress responses and intracellular signaling pathways that can culminate in apoptosis. The mechanisms described are detailed in Table 3. Furthermore, genetic engineering allows for the modification of bacteria to produce “suicide” enzymes (suicide genes). For instance, bacteria can be programmed to produce cytosine deaminase, which converts an administered prodrug into a toxic drug (e.g., 5-fluorocytosine into 5-fluorouracil) locally within the tumor, resulting in high drug concentrations within the tumor with minimal exposure to healthy tissue. Alternatively, other approaches utilize bacteria to produce enzymes such as nitroreductases, which activate prodrugs exclusively within the tumor [95,98].

The presence of bacteria within a tumor has been demonstrated to modify the microenvironment. For instance, bacteria can degrade the extracellular matrix using proteolytic enzymes, which improves the penetration of chemotherapeutic drugs. This approach allows for combination therapy, which can increase treatment efficiency [94,125]. A notable benefit of oncolytic bacteria is their capacity to potently stimulate the immune system. Natural and engineered bacteria have been shown to act as living adjuvants, stimulating both the innate immune response (NK cells, neutrophils and macrophages) and the adaptive response (T lymphocytes). The structural components of bacteria, such as LPS, flagellin, peptidoglycans, and other pathogen-associated molecular patterns (PAMPs), have been shown to activate Toll-like receptors (TLRs) and other signaling pathways. This results in the production of proinflammatory cytokines (e.g., TNF-α and IL-1β) and chemokines, which in turn recruit immune cells to the tumor site. Consequently, a strong antitumor immune response can be initiated. Cytotoxic T lymphocytes (CD8^+^), NK cells, and macrophages have the capacity to recognize and destroy cancer cells [92,93]. Furthermore, bacteria can function as vectors to deliver tumor-associated antigens (TAAs), which, in turn, can stimulate a specific immune response against the tumor [97]. A selection of bacteria with immunomodulatory capabilities is presented in Table 4, along with their respective effects on tumors.

Another type of cancer therapy that is attracting researchers’ attention is bacteriobot therapy, which uses bacteria-based microrobots [154]. These systems are emerging as a novel class of biohybrid systems that integrate living microorganisms with engineered components to achieve active, targeted drug delivery [155,156]. Unlike conventional chemotherapy, which relies on passive diffusion and often results in low therapeutic agent accumulation at the disease site, bacteriobots exploit bacteria’s intrinsic motility, chemotaxis, and environmental sensing capabilities of bacteria to navigate complex biological environments and selectively localize within pathological tissues, particularly solid tumors [157,158,159]. Their ability his tumor-targeting ability is largely driven by characteristic features of the TME, such as hypoxia, necrosis, and abnormal vascularization, which create favorable niches for bacterial colonization [157,159].

In the context of oncology, bacteriobots offer several unique therapeutic advantages. It has been demonstrated that certain bacterial strains, including attenuated *Salmonella* and *Clostridium*, exhibit natural tropism toward hypoxic tumor cores. These regions are typically resistant to conventional therapies such as radiotherapy and chemotherapy [101]. The exploitation of this property enables bacteriobots to penetrate deeply into tumor tissue and deliver therapeutic payloads directly to otherwise inaccessible regions [154]. Engineered bacteria can be programmed using synthetic biology approaches to produce and release anticancer agents in situ, including cytotoxic proteins, prodrug-converting enzymes, or immune-modulating factors. This approach enables localized therapy with significantly reduced systemic exposure and toxicity [160,161].

Furthermore, bacteriobots have the potential to play a crucial role in cancer immunotherapy. The presence of bacteria within tumors has been demonstrated to stimulate innate and adaptive immune responses. This stimulation is achieved by activating pattern recognition receptors and promoting the recruitment of immune cells. Engineered bacteria can be designed to secrete cytokines or TAAs, thereby enhancing anti-tumor immunity and potentially overcoming immunosuppressive TME. This positions bacteriobots as a promising platform for combination therapies, integrating targeted drug delivery with immune system activation [162,163].

Another significant aspect is the potential for external control and guidance. For instance, magnetotactic bacteriobots can be directed using external magnetic fields, leading to enhanced tumor targeting precision and distribution within the tumor mass. This degree of control may enable the real-time adjustment of treatment, with the potential to enhance therapeutic efficacy while minimizing damage to healthy tissues [164,165,166].

Despite these encouraging advancements, several challenges must be addressed before clinical implementation becomes standard practice. These include ensuring precise control over bacterial proliferation, preventing unintended infections, managing host immune responses, and achieving reproducible large-scale production [155,167]. Nevertheless, ongoing advances in the fields of genetic engineering, biomaterials, and microrobotics continue to push the field forward, suggesting that bacteriobots could become a transformative modality in future oncological therapies, particularly for hard-to-treat and drug-resistant tumors [168].

## 4. Limitations and Challenges

Despite the significant therapeutic potential of oncolytic bacteria and bacteriobots, recent literature suggests certain limitations to the use of this method, including biological, safety and translational challenges, which currently limit their wider clinical application in human and veterinary oncology [169]. The majority of extant evidence originates from murine tumor models, where studies involving companion animals, particularly dogs and cats, remain limited [149,170]. Consequently, the efficacy and safety observed in experimental systems may not fully translate into veterinary clinical practice.

A significant concern related to bacterial therapies is biosafety. Despite the use of attenuated or genetically modified bacterial strains in cancer therapy to reduce virulence, there are still potential risks associated with uncontrolled bacterial proliferation, off-target colonization, bacteremia, and septic complications remain potential risks [169]. Gram-negative bacteria such as *S. typhimurium* have been observed to induce excessive inflammatory responses due to LPS release. These responses can potentially lead to systemic toxicity, a condition known as a cytokine storm, or septic shock [171]. Similarly, bacterial persistence in healthy tissues may result in unintended tissue damage or chronic inflammation [169]. These safety concerns are of particular importance in immunocompromised patients and animals receiving concurrent chemotherapy.

Another important limitation is the host immune response against therapeutic bacteria. The mononuclear phagocyte system, particularly the macrophages located in the liver and spleen, plays a crucial role in the clearance of bacteria within tumors following systemic administration, potentially mitigating bacterial accumulation [128,169]. In veterinary patients, species-specific differences in innate and adaptive immunity between dogs, cats, and humans may additionally influence bacterial survival, tumor colonization efficiency, and treatment outcomes [96]. Furthermore, the presence of pre-existing immunity and neutralizing antibodies has been demonstrated to impair the repeated administration of bacterial therapeutics [169].

The efficacy of numerous oncolytic bacterial therapies is significantly influenced by the presence of hypoxic and necrotic tumor regions [98]. Consequently, highly vascularized or early-stage tumors lacking substantial necrosis may be less susceptible to bacterial colonization [141]. To address this limitation, current research efforts are directed towards engineering bacteria capable of responding to alternative tumor-associated signals, including but not limited to acidic pH, lactate concentration, ROS, quorum-sensing molecules, or specific metabolic pathways [96]. It has been demonstrated that facultative anaerobic bacteria, including *Salmonella* and genetically modified *Escherichia coli*, may exhibit partial activity in regions of less hypoxic tumors [96,172]. Additionally, synthetic biology methodologies enable the construction of programmable genetic circuits that regulate bacterial proliferation and therapeutic gene expression in response to tumor-specific environmental cues [141].

Additional concerns relate to the use of genetically modified microorganisms. Horizontal gene transfer between engineered bacteria and native microbiota remains a theoretical but important biosafety issue, particularly regarding the dissemination of antibiotic resistance genes or synthetic genetic constructs into the environment [141,169]. Regulatory agencies require extensive biosafety validation, environmental risk assessment, and strict containment strategies before granting approval to genetically engineered bacterial therapeutics [169]. These regulatory challenges are particularly pronounced in the field of veterinary medicine, where the approval pathways for genetically modified live biotherapeutics remain inadequately established [96,173].

In addition, bacteriobots encounter numerous technological and translational limitations. The efficacy of these treatments may be limited by various factors, including immune clearance, heterogeneous tumor architecture, abnormal blood flow, and limited penetration into dense tumor tissue [96,141,169]. In dogs and cats, rapid recognition and elimination by phagocytic cells may substantially reduce circulation time and therapeutic efficacy [96]. Furthermore, the large-scale manufacturing, standardization, reproducibility, and long-term biosafety of biohybrid microrobotic systems have yet to be adequately characterized [96]. The potential of external guidance systems, including magnetic navigation, has been demonstrated in preclinical studies. However, further optimization is necessary before their routine veterinary application becomes feasible [141,169].

To date, the number of studies evaluating bacterial therapies in spontaneous tumors occurring in companion animals such as dogs and cats remains limited [96,169]. The majority of available data originates from murine models, which do not fully recapitulate the complexity, heterogeneity, microbiota composition, or immune responses observed in dogs and cats [96,141]. Simultaneously, it is emphasized that dogs and cats spontaneously develop cancers that closely resemble human malignancies, and that their microbiome and immune systems are in many respects more similar to humans than those of traditional mouse models. Additionally, they share the human environment, making them valuable models in comparative oncology. Consequently, data derived from both animal and human models remain largely hypothetical within the context of veterinary applications, requiring further integration through translational research to achieve clinical relevance. Consequently, further veterinary clinical studies are required to determine optimal dosing strategies, long-term safety, therapeutic efficacy, and regulatory frameworks for the future implementation of bacterial therapies in veterinary oncology.

The stimulation of innate and adaptive immune responses by oncolytic bacteria constitutes one of the principal mechanisms underlying their antitumor efficacy. However, excessive systemic immune activation may also increase the risk of systemic inflammatory response syndrome (SIRS), particularly in geriatric veterinary patients characterized by immunosenescence, chronic low-grade inflammation (“inflammaging”), and reduced cardiopulmonary and renal reserve [172,174,175]. The recognition of bacterial PAMPs, including LPS and lipoteichoic acid, by Toll-like receptors induces the release of pro-inflammatory cytokines such as TNF-α, IL-1β, and IL-6. This, in turn, may contribute to systemic cytokine dysregulation, endothelial injury, hypotension, and secondary organ dysfunction when activation becomes uncontrolled. Excessive neutrophil extracellular trap (NET) formation has additionally been associated with tissue injury, sepsis-like syndromes, and amplified inflammatory responses. These mechanisms are of particular relevance in older veterinary oncology patients due to age-associated alterations in innate immune regulation and reduced physiologic resilience [176,177].

Current strategies that have been proposed to mitigate off-target inflammatory activation include attenuation of bacterial virulence, tumor-restricted colonization, inducible gene-expression systems, and the incorporation of synthetic biology approaches that enable environmental sensing and conditional therapeutic activation. Genetically modified strains with reduced pathogenicity, including attenuated *S. typhimurium* and *Clostridium novyi*-NT, have demonstrated improved safety profiles while preserving antitumor activity. Additional approaches include auxotrophic engineering, which restricts bacterial survival to nutrient-enriched TMEs, quorum-sensing circuits, which control bacterial lysis; and externally regulated promoters, which enable temporal control of therapeutic payload release [149]. Combination strategies involving localized delivery, dose de-escalation, antibiotic “kill switches,” and integration with immune checkpoint modulation have also been proposed to reduce systemic toxicity while maintaining immunostimulatory efficacy. In veterinary settings, the provision of support by management may require the implementation of monitoring protocols analogous to those employed in the context of SIRS and sepsis. Such monitoring may include surveillance for pyrexia, hypotension, coagulopathy, and early organ dysfunction [178,179].

## 5. Conclusions and Perspectives

A growing body of research suggests that disrupted microbiota composition may play a significant role in fostering cancer development within the canine and feline populations [20]. Therefore, a comprehensive understanding of the drivers of dysbiosis is imperative for both prevention and innovation.

This comprehensive understanding is not only instrumental in preventing the condition but also paves the way for the development of novel therapeutic interventions that utilize bacteria as active agents in the combat against malignancy. In this context, oncolytic bacteria emerge as a promising, though still poorly understood, therapeutic tool in veterinary oncology. Their capacity to selectively colonize tumor tissues, induce an immune response, and also affect metastases makes them a valuable future alternative or complement to conventional treatment methods such as chemotherapy and radiotherapy in animals [172,180]. Despite the challenges associated with the optimization of delivery methods and the management of adverse effects, further research could potentially position oncolytic bacteria as a crucial element of future veterinary oncology, contributing to enhanced quality and length of life for animal patients.

This concept can be further investigated through the lens of oncological engineering. In particular, the potential for realizing the ideal oncological tool—a device capable of selective tumor targeting and localized drug delivery without off-target toxicity—through bacteriobots is promising [181]. In contrast to conventional nanoparticles, biohybrid systems utilize natural bacterial chemotaxis to navigate the complex TME, actively reaching necrotic and hypoxic cores that are inaccessible to standard therapies [158].

A comprehensive investigation is imperative to understand the causes and systemic effects of dysbiosis in companion animals. This investigation should also explore its influence on overall health and the increased risk of chronic disease. As demonstrated in previous research, pathogenic bacteria should not be viewed solely as biological threats; rather, through advanced molecular engineering, their intrinsic properties can be repurposed. By harnessing and enhancing their innate biological mechanisms, such as selective tissue colonization and immune activation, these organisms can be transformed into potent therapeutic agents with significant anti-cancer potential.

## Figures and Tables

**Table 2:** Correlations between microbiota disorders and the development or course of cancer in dogs and cats.

(**A**) **Cats**					
**Clinical Condition/** **Disease Model**	**Tumor Location**	**Microbiota Alterations**	**Observed Clinical Correlations**	**Pathogenetic Significance**	**Reference**
Low-grade intestinal lymphoma	Small intestine	↓ diversity; ↑ *Proteobacteria*	Microbiota profile similar to IBD; frequent history of chronic enteropathy	Similar microbiota profiles suggest overlapping pathophysiology with IBD	[76]
IBD	Small intestine	Altered *Firmicutes*/ *Bacteroidetes* ratio	Subset of patients later developed intestinal lymphoma	Dysbiosis as a potential initiating factor for neoplastic transformation	[25]
Intestinal lymphoma (various grades)	Small intestine	Microbiota similar to IBD	Diagnostic overlap; gradual progression	Microbiota as a potential biomarker differentiating inflammation and neoplasia	[76]
(**B**) **Dogs**					
**Clinical Condition/** **Disease Model**	**Tumor Location**	**Microbiota Alterations**	**Observed Clinical Correlations**	**Pathogenetic Significance**	**Reference**
High-grade intestinal lymphoma	Small intestine	Severe dysbiosis; ↑ *Enterobacteriaceae*	More severe clinical signs and rapid disease progression	Degree of dysbiosis correlates with tumor aggressiveness	[54]
Gastrointestinal tumors (various histotypes)	Gastrointestinal tract	Altered gut metabolome; ↓ SCFA levels	Weight loss, hypoalbuminemia	Metabolic TME promoting progression	[78]
Multicentric lymphoma	Lymphatic system	Gut dysbiosis despite absence of intestinal tumors	Correlation with systemic inflammatory markers	Systemic immunometabolic effects of gut microbiota	[52]
Cancers treated with chemotherapy	Multiple sites	Variable degree of pre-treatment dysbiosis	Better chemotherapy tolerance in dogs with lower dysbiosis	Microbiota as a predictive factor of therapeutic response	[79]
Cutaneous tumors with chronic inflammation	Skin	Local skin dysbiosis (case reports)	Persistent local inflammation	Possible local promotion of carcinogenesis	[80]

**Table 3:** Selected bacteria exhibiting direct oncolytic effect and associated immune activation.

Bacteria	Mechanisms of Direct Oncolysis	Secondary Immune Activation	Oncolytic Outcomes	References
*Salmonella typhimurium*	- Preferential tumor colonization (hypoxic regions, tumor metabolites) - Bacterial cytotoxicity - Tumor blood vessel damage via TNF-α - Tumor cell lysis (similar to immunogenic cell death)	- Activation of TLR4/TLR5 → IL-1β, IL-12, TNF-α - Recruitment of neutrophils, macrophages, Natural Killer cells (NK cells) - Activation of CD8^+^ T cells - Reduction of MDSCs and Tregs in TME	- Direct tumor destruction - Improved immune infiltration - Activation of adaptive immunity - Remodeling TME toward pro-inflammatory state	[108,109,110,111,112,113]
*Listeria monocytogenes*	- Intracellular infection, vector for tumor antigens - Induction of mitochondrial stress/Reactive Oxygen Species (ROS) in tumor cells	- Activation of dendritic cells (DCs), antigen presentation via MHC I/II - Strong CD8^+^ T cell response - Th1 bias (IL-12 → IFN-γ) - Remodeling of TME	- Facilitates tumor antigen recognition - Stimulates cytotoxic T cell response - Enhances adaptive immunity	[114,115,116,117,118,119]
*Clostridium novyi*-NT	- Spore germination in hypoxic tumor areas - Production of lytic enzymes (lipases, phospholipases, proteases) → tumor necrosis	- Local inflammation: recruitment of neutrophils, macrophages - Release of tumor antigens → CD8^+^ T cell activation - TME shift toward pro-inflammatory, immunogenic state - Synergy with checkpoint inhibitors	- Massive tumor necrosis - Recruitment and activation of immune cells - TME remodeling toward immunogenic phenotype	[98,120,121,122,123,124]

**Table 4:** Selected bacteria primarily acting through immunomodulation and antitumor immune enhancement.

Bacteria	Immune Signaling Pathways	Immunomodulatory Mechanisms	Immune-Mediated Antitumor Effects	References
*Bifidobacterium*	- DC maturation (CD103^+^ DC) - Enhanced CD8^+^ T cell activation - SCFA signaling (butyrate, acetate) - Reduced Treg/MDSC	- Increases antigen presentation efficiency - Enhances Interferon-gamma (IFN-γ) production - Boosts response to PD-1/PD-L1 blockade - Promotes M1 macrophage polarization	- Stronger cytotoxic CD8^+^ T-cell responses - Improved checkpoint inhibitor efficacy - Reduced tumor immunosuppression	[126,127,128,129,130,131,132,133]
*Escherichia coli*	- TLR4 (LPS) activation - TLR5 (flagellin) - Activation of DC, NK, and macrophages - Engineered bacterial payload delivery	- Strong innate immune activation (TNF-α, IL-12, IL-6) - Engineered release of IL-2, IL-12, Stimulator of Interferon Genes (STING) agonists - OMV-based cancer vaccination - Tumor colonization enables targeted delivery	- Potent innate immune stimulation - Induction of CD8^+^ T-cell responses - Enhanced tumor recognition through Outer Membrane Vesicles (OMVs) - Effective microbial delivery system in tumors	[134,135,136,137]
*Mycobacterium bovis* (BCG)	- TLR2, TLR4, NOD2 - Th1 polarization (IFN-γ, IL-2) - Trained immunity (epigenetic reprogramming)	- Strong activation of macrophages, neutrophils, DC - Induces IL-12, TNF-α, IL-1β - Enhances antigen presentation (MHC I/II) - Recruits immune cells into TME	- Clinically validated immune stimulation in bladder cancer - Converts “cold” tumors to “hot” - Long-term enhancement of innate immunity	[138,139,140,141,142,143,144,145,146,147]
*Serratia marcescens*	- Prodigiosin-mediated modulation - M1 macrophage activation - Reduction of Tregs & MDSCs	- Induces immunogenic apoptosis via mitochondrial damage - Promotes inflammatory cytokines (IL-12, TNF-α) - Reduces immunosuppressive cell populations - Enhances DC function	- Reversal of TME immunosuppression - Increased T-cell priming - Higher antigen release and recognition	[148,149,150,151,152,153]

## Data Availability

No new data were created or analyzed in this study. Data sharing is not applicable to this article.

## Abbreviations

The following abbreviations are used in this manuscript:

AhR	Aryl hydrocarbon receptor
BCFA	Branched-chain fatty acids
CagA protein	Cytotoxin-associated gene A protein
CIE	Chronic inflammatory enteropathy
DC	Dendritic cell
FMT	Fecal microbiota transplantation
GALT	Gut-associated lymphoid tissue
IARC	International Agency for Research on Cancer
IBD	Inflammatory Bowel Disease
IFN-γ	Interferon-gamma
IL-1β	Interleukin-1 beta
IL-2	Interleukin-2
IL-6	Interleukin-6
IL-12	Interleukin-12
LGITL	Low-grade intestinal T-cell lymphoma
LPS	Lipopolysaccharide
MALT-type	Mucosa-Associated Lymphoid Tissue–type
MDSC	Myeloid-Derived Suppressor Cells
MHC	Major Histocompatibility Complex
NET	Neutrophil Extracellular Trap
NF-κB	Nuclear factor-κB
NHPH	non-Helicobacter pylori Helicobacters
NK cells	Natural Killer cells
NOD2	Nucleotide-binding oligomerization domain 2
OMVs	Outer Membrane Vesicles
PAMPs	Pathogen-associated molecular patterns
PD-1	Programmed cell death protein 1
PD-L1	Programmed Death-Ligand 1
ROS	Reactive Oxygen Species
SCC	Squamous Cell Carcinoma
SCFAs	Short-chain fatty acids
SIRS	Systemic Inflammatory Response Syndrome
STING	Stimulator of Interferon Genes
TAAs	Tumor-associated antigens
Th1	T helper 1
Th17	T helper 17
TME	Tumor microenvironment
TNF-α	Tumor Necrosis Factor-alpha
TLR2	Toll-like receptor 2
TLR4	Toll-like receptor 4
TLR5	Toll-like receptor 5
TLRs	Toll-like receptors
Treg	Regulatory T cells
VacA toxin	Vacuolating cytotoxin A

## References

[1] Fine A.H., Ng Z., Knight M.P., Griffin T.C., Braun L., Feldman S. (2025). A commentary on perspectives of the pet effect: Exploring public awareness, community impact, and public policy implications. Pets.

[2] van der Weyden L. (2025). Focus on tumours in pet animals. Vet. Sci..

[3] Ahn S., Yun J.-H. (2026). Comparative Cancer Genetics and Veterinary Therapeutics in Dogs and Cats: A Species-Aware Framework for Comparative Oncology. Life.

[4] Rupasinghe R., Cao J.M.D., Kent M., Lejeune A.T., Rebhun R.B., Martínez-López B. (2026). Descriptive epidemiology of canine and feline cancer in California, United States from 2000 to 2019. Vet. J..

[5] Mirowski A. (2012). Żywienie w profilaktyce i leczeniu chorób nowotworowych psów i kotów. Cz. I. Wiadomości wstępne. Mag. Wet..

[6] Di Cerbo A., Palmieri B., De Vico G., Iannitti T. (2014). Onco-epidemiology of domestic animals and targeted therapeutic attempts: Perspectives on human oncology. J. Cancer Res. Clin. Oncol..

[7] Śmiech A., Ślaska B., Łopuszyński W., Jasik A., Szczepanik M., Wilkołek P. (2017). Epidemiological study of canine mast cell tumours according to the histological malignancy grade. Pol. J. Vet. Sci..

[8] Sapierzyński R., Badowska-Kozakiewicz A., Malicka E. (2008). Nowotwory układu pokarmowego psów i kotów. Retrospektywny przegląd 87 przypadków. Med. Weter..

[9] Śmiech A., Bulak K., Łopuszyński W., Puła A. (2023). Incidence and the risk of occurrence of benign and malignant canine skin tumours in Poland—A five-year retrospective study. J. Vet. Res..

[10] Thompson K.G., Pool R.R., Meuten J. (2002). Tumors of Bones. Tumors in domestic animals.

[11] Bertram C.A., Donovan T.A., Bartel A. (2024). Mitotic activity: A systematic literature review of the assessment methodology and prognostic value in canine tumors. Vet. Pathol..

[12] Khan A.A., Sirsat A.T., Singh H., Cash P. (2021). Microbiota and cancer: Current understanding and mechanistic implications. Clin. Transl. Oncol..

[13] Gevers D., Kugathasan S., Denson L.A., Vázquez-Baeza Y., Van Treuren W., Ren B., Schwager E., Knights D., Song S.J., Yassour M. (2014). The Treatment-Naive Microbiome in New-Onset Crohn’s Disease. Cell Host Microbe.

[14] Lloyd-Price J., Arze C., Ananthakrishnan A.N., Rahnavard A., Schirmer M., Ávila-Pacheco J., Poon T., Andrews E., Ajami N.J., Bonham K.S. (2019). Multi-omics of the gut microbial ecosystem in inflammatory bowel diseases. Nature.

[15] Turnbaugh P.J., Ley R.E., Mahowald M.A., Magrini V., Mardis E.R., Gordon J.I. (2006). An obesity-associated gut microbiome with increased capacity for energy harvest. Nature.

[16] Gilbert J.A., Quinn R.A., Debelius J., Xu Z.Z., Morton J., Garg N., Jansson J.K., Dorrestein P.C., Knight R. (2016). Microbiome-wide association studies link dynamic microbial consortia to disease. Nature.

[17] Carding S., Verbeke K., Vipond D.T., Corfe B.M., Owen L.J. (2015). Dysbiosis of the gut microbiota in disease. Microb. Ecol. Health Dis..

[18] DeGruttola A.K., Low D., Mizoguchi A., Mizoguchi E. (2016). Current understanding of dysbiosis in disease in human and animal models. Inflamm. Bowel Dis..

[19] Gut Microbial Dysbiosis. https://onehealth.colostate.edu/2022/02/03/gut-microbial-dysbiosis/.

[20] Filippo D., Guardone L., Listorti V., Elisabetta R. (2024). Microbiome in Cancer: A comparative analysis between humans and dogs. Vet. J..

[21] Aluai-Cunha C.S., Pinto C.A., Correia I.A.D.F.L., Dos Reis Serra C.A., Santos A.A.F. (2023). The animal’s microbiome and cancer: A translational perspective. Vet. Comp. Oncol..

[22] Pilla R., Suchodolski J.S. (2020). The role of the canine gut microbiome and metabolome in health and gastrointestinal disease. Front. Vet. Sci..

[23] Horodyska I., Kasperska P., Michalski K., Bubak J., Herman I., Miszczak M. (2025). Natural microbiota of dogs and cats as a source and vector of resistance genes—Clinical significance. Int. J. Mol. Sci..

[24] Pereira A.M., Clemente A. (2021). Dogs’ Microbiome from Tip to Toe. Top. Companion Anim. Med..

[25] Suchodolski J.S. (2011). Companion animals symposium: Microbes and gastrointestinal health of dogs and cats. J. Anim. Sci..

[26] Whittle M.J., Castillo-Fernandez J., Amos G.C.A., Watson P. (2024). Metagenomic characterisation of canine skin reveals a core healthy skin microbiome. Sci. Rep..

[27] Banchi P., Spanoghe L., Maes D., Morrell J., Van Soom A. (2024). The reproductive microbiome in dogs: Friend or foe?. Vet. J..

[28] Suchodolski J.S. (2021). Analysis of the gut microbiome in dogs and cats. Vet. Clin. Pathol..

[29] Gao Y., Fang Y., Huang Y., Ma R., Chen X., Wang F., Pei X., Gao Y., Chen X., Liu X. (2022). MIIP functions as a novel ligand for ITGB3 to inhibit angiogenesis and tumorigenesis of triple-negative breast cancer. Cell Death Dis..

[30] Parida S., Wu S., Siddharth S., Wang G., Muniraj N., Nagalingam A., Hum C., Mistriotis P., Hao H., Talbot C.C. (2021). A Procarcinogenic Colon Microbe Promotes Breast Tumorigenesis and Metastatic Progression and Concomitantly Activates Notch and β-Catenin Axes. Cancer Discov..

[31] Ji X., Li R., Hu X., Tian Y., Liu L., Zhang C., Xu L., Chen Y., Xie H., Mao L. (2023). Construction of model animals to explore intestinal microbiome for detection of breast cancer. PLoS ONE.

[32] Li Q. (2023). Bacterial infection and microbiota in carcinogenesis and tumor development. Front. Cell. Infect. Microbiol..

[33] Plewa P., Kiełbowski K., Mentel O., Figiel K., Bakinowska E., Becht R., Banach B., Pawlik A. (2025). Bacteria and carcinogenesis and the management of cancer: A narrative review. Pathogens.

[34] Breczko W.J., Bubak J., Miszczak M. (2024). The Importance of Intestinal Microbiota and Dysbiosis in the Context of the Development of Intestinal Lymphoma in Dogs and Cats. Cancers.

[35] Singh G., Chaudhry Z., Boyadzhyan A., Sasaninia K., Rai V. (2025). Dysbiosis and colorectal cancer: Conducive factors, biological and molecular role, and therapeutic prospectives. Explor. Target. Anti-Tumor Ther..

[36] Rowe J.C., Winston J.A. (2024). Collaborative Metabolism: Gut Microbes Play a Key Role in Canine and Feline Bile Acid Metabolism. Vet. Sci..

[37] Duizer C., De Zoete M.R. (2023). The Role of Microbiota-Derived Metabolites in Colorectal Cancer. Int. J. Mol. Sci..

[38] Aluai-Cunha C., Oliveira D., Gregório H., Petrucci G., Correia A., Serra C., Santos A. (2025). Characterisation of the faecal microbiota in dogs with mast cell tumours compared with healthy dogs. Animals.

[39] Xie Z., Wu Z., Liu Y., Gu Y., Niu J., Lv K. (2025). Intratumoral microbiota: Implications for cancer progression and treatment. Front. Microbiol..

[40] Xu K., Motiwala Z., Corona-Avila I., Makhanasa D., Alkahalifeh L., Khan M.W. (2025). The gut microbiome and its multifaceted role in cancer metabolism, initiation, and progression: Insights and therapeutic implications. Technol. Cancer Res. Treat..

[41] Liu Q., Yang Y., Pan M., Yang F., Yu Y., Qian Z. (2024). Role of the gut microbiota in tumorigenesis and treatment. Theranostics.

[42] Ivleva E.A., Grivennikov S.I. (2022). Microbiota-driven mechanisms at different stages of cancer development. Neoplasia.

[43] Drut A., Mkaouar H., Kriaa A., Mariaule V., Akermi N., Méric T., Sénécat O., Maguin E., Hernandez J., Rhimi M. (2024). Gut microbiota in cats with inflammatory bowel disease and low-grade intestinal T-cell lymphoma. Front. Microbiol..

[44] Zhang S., Paul S., Kundu P. (2022). NF-ΚB Regulation by gut microbiota decides homeostasis or disease outcome during ageing. Front. Cell Dev. Biol..

[45] Peña-Durán E., García-Galindo J.J., López-Murillo L.D., Huerta-Huerta A., Balleza-Alejandri L.R., Beltrán-Ramírez A., Anaya-Ambriz E.J., Suárez-Rico D.O. (2025). Microbiota and inflammatory markers: A review of their interplay, clinical implications, and metabolic disorders. Int. J. Mol. Sci..

[46] Moon C.D., Young W., Maclean P.H., Cookson A.L., Bermingham E.N. (2018). Metagenomic insights into the roles of *Proteobacteria* in the gastrointestinal microbiomes of healthy dogs and cats. MicrobiologyOpen.

[47] Kane A.B., Stewart B.W., Straif K. (2019). Inflammation. Tumour Site Concordance and Mechanisms of Carcinogenesis.

[48] Questa M., Weimer B.C., Fiehn O., Chow B., Hill S.L., Ackermann M.R., Lidbury J.A., Steiner J.M., Suchodolski J.S., Marsilio S. (2024). Unbiased serum metabolomic analysis in cats with naturally occurring chronic enteropathies before and after medical intervention. Sci. Rep..

[49] Asgharzadeh S., Pourhajibagher M., Bahador A. (2025). The microbial landscape of tumors: A deep dive into intratumoral microbiota. Front. Microbiol..

[50] Zhao Y., Yang Z., Wu D., Zhao H. (2025). Dissecting the intratumoral microbiome landscape in lung cancer. Front. Immunol..

[51] Bae H., Lim S.K., Jo H.E., Oh Y., Park J., Choi H.-J., Yu D. (2023). Fecal microbiome in dogs with lymphoid and nonlymphoid tumors. J. Vet. Intern. Med..

[52] Gavazza A., Rossi G., Lubas G., Cerquetella M., Minamoto Y., Suchodolski J.S. (2017). Faecal microbiota in dogs with multicentric lymphoma. Vet. Comp. Oncol..

[53] Rowaiye A., Ibeanu G.C., Bur D., Nnadi S., Mgbeke O.E., Morikwe U. (2024). Gut microbiota alteration—Cancer relationships and synbiotic roles in cancer therapies. Microbe.

[54] Omori M., Maeda S., Igarashi H., Ohno K., Sakai K., Yonezawa T., Horigome A., Odamaki T., Matsuki N. (2017). Fecal microbiome in dogs with inflammatory bowel disease and intestinal lymphoma. J. Vet. Med. Sci..

[55] Almonte A.A., Thomas S., Iebba V., Kroemer G., Derosa L., Zitvogel L. (2026). Gut dysbiosis in oncology: A risk factor for immunoresistance. Cell Res..

[56] He R., Qi P., Shu L., Ding Y., Zeng P., Wen G., Xiong Y., Deng H. (2025). Dysbiosis and extraintestinal cancers. J. Exp. Clin. Cancer Res..

[57] Barko P.C., Williams D.A., Wu Y.-A., Steiner J.M., Suchodolski J.S., Gal A., Marsilio S. (2023). Chronic Inflammatory Enteropathy and Low-Grade Intestinal T-Cell Lymphoma Are Associated with Altered Microbial Tryptophan Catabolism in Cats. Animals.

[58] Chen H., Yu S., Zhang M., Tian B., Yang L., Lu J. (2026). Gut microbial metabolites in inflammatory bowel disease: Immunological mechanisms regulating Treg/Th17 balance and therapeutic potential. Front. Immunol..

[59] Ma W.-W., Huang Z.-Q., Liu K., Li D.-Z., Mo T.-L., Liu Q. (2024). The role of intestinal microbiota and metabolites in intestinal inflammation. Microbiol. Res..

[60] Chen Y., Chen Y.-X. (2021). Microbiota-Associated Metabolites and Related Immunoregulation in Colorectal Cancer. Cancers.

[61] Keane J.M., Walsh C.J., Cronin P., Baker K., Melgar S., Cotter P.D., Joyce S.A., Gahan C.G.M., Houston A., Hyland N.P. (2022). Investigation of the gut microbiome, bile acid composition and host immunoinflammatory response in a model of azoxymethane-induced colon cancer at discrete timepoints. Br. J. Cancer.

[62] Li G., Xiong Y., Li Z., Yu Q., Li S., Xie J., Zeng S., Yu D., Yang Y., Yu J. (2025). Gut microbiota-derived metabolites modulate Treg/Th17 balance: Novel therapeutic targets in autoimmune diseases. Front. Immunol..

[63] Tian X., Yu L., Shi J., Zhao R., Shi R., Zhang Y., Zhao J., Tian C., Cui H., Guan H. (2023). Advances in the role of gut microbiota in the regulation of the tumor microenvironment (Review). Oncol. Rep..

[64] Farhadi Rad H., Tahmasebi H., Javani S., Hemati M., Zakerhamidi D., Hosseini M., Alibabaei F., Banihashemian S.Z., Oksenych V., Eslami M. (2024). Microbiota and Cytokine Modulation: Innovations in Enhancing Anticancer Immunity and Personalized Cancer Therapies. Biomedicines.

[65] Rodrigues R.R., Karumuru V., Nuss S., Elliott M., Shriver I., Chao C.-M., Berriatua R.C., Doyle H.A., Tripp C., Mamula M.J. (2025). Gut microbiota of dogs with cancer receiving anti-EGFR/HER2 immunization reveals potential biomarkers of patient survival. Vet. Oncol..

[66] Doocey C.M., Finn K., Murphy C., Guinane C.M. (2022). The impact of the human microbiome in tumorigenesis, cancer progression, and biotherapeutic development. BMC Microbiol..

[67] Duan Y., Xu Y., Dou Y., Xu D. (2025). *Helicobacter pylori* and gastric cancer: Mechanisms and new perspectives. J. Hematol. Oncol..

[68] Hooi J.K.Y., Lai W.Y., Ng W.K., Suen M.M.Y., Underwood F.E., Tanyingoh D., Malfertheiner P., Graham D.Y., Wong V.W.S., Wu J.C.Y. (2017). Global prevalence of *Helicobacter pylori* infection: Systematic review and meta-analysis. Gastroenterology.

[69] IARC Working Group on the Evaluation of Carcinogenic Risks to Humans (2012). A Review of Human Carcinogens: Part B: Biological Agents.

[70] Yong X., Tang B., Li B.S., Xie R., Hu C.J., Luo G., Qin Y., Dong H., Yang S.M. (2015). *Helicobacter pylori* virulence factor CagA promotes tumorigenesis of gastric cancer via multiple signaling pathways. Cell Commun. Signal..

[71] Valenzuela M.A. (2015). *Helicobacter pylori*-induced inflammation and epigenetic changes during gastric carcinogenesis. World J. Gastroenterol..

[72] Taillieu E., Chiers K., Amorim I., Gärtner F., Maes D., Van Steenkiste C., Haesebrouck F. (2022). Gastric *Helicobacter* species associated with dogs, cats and pigs: Significance for public and animal health. Vet. Res..

[73] Helicobacter Infection in Small Animals. https://www.msdvetmanual.com/digestive-system/diseases-of-the-stomach-in-small-animals/helicobacter-infection-in-small-animals.

[74] Bridgeford E.C., Marini R.P., Feng Y., Parry N.M.A., Rickman B., Fox J.G. (2008). Gastric *Helicobacter* species as a cause of feline gastric lymphoma: A viable hypothesis. Vet. Immunol. Immunopathol..

[75] Wu M., Tian C., Zou Z., Jin M., Liu H. (2024). Gastrointestinal microbiota in gastric cancer: Potential mechanisms and clinical applications—A literature review. Cancers.

[76] Marsilio S., Pilla R., Sarawichitr B., Chow B., Hill S.L., Ackermann M.R., Estep J.S., Lidbury J.A., Steiner J.M., Suchodolski J.S. (2019). Characterization of the fecal microbiome in cats with inflammatory bowel disease or alimentary small cell lymphoma. Sci. Rep..

[77] Lamas B., Natividad J.M., Sokol H. (2018). Aryl Hydrocarbon Receptor and Intestinal Immunity. Mucosal Immunol..

[78] Kaga C., Kakiyama S., Hokkyo A., Ogata Y., Shibata J., Nagahara T., Nakazawa M., Nakagawa T., Tsujimoto H., Chambers J.K. (2024). Characterization of faecal microbiota and serum inflammatory markers in dogs diagnosed with chronic enteropathy or small-cell lymphoma: A pilot study. Sci. Rep..

[79] Jugan M.C., Wouda R.M., Higginbotham M.L. (2021). Preliminary evaluation of probiotic effects on gastrointestinal signs in dogs with multicentric lymphoma undergoing multi-agent chemotherapy: A randomised, placebo-controlled study. Vet. Rec. Open.

[80] Bromfield J.I., Zaugg J., Straw R.C., Cathie J., Krueger A., Sinha D., Chandra J., Hugenholtz P., Frazer I.H. (2024). Characterization of the skin microbiome in normal and cutaneous squamous cell carcinoma affected cats and dogs. mSphere.

[81] Wang J., Zhu N., Su X., Gao Y., Yang R. (2023). Gut-Microbiota-Derived Metabolites Maintain Gut and Systemic Immune Homeostasis. Cells.

[82] Spivak I., Fluhr L., Elinav E. (2022). Local and Systemic Effects of Microbiome-derived Metabolites. EMBO Rep..

[83] Wang M., Zhang L., Chang W., Zhang Y. (2023). The Crosstalk between the Gut Microbiota and Tumor Immunity: Implications for Cancer Progression and Treatment Outcomes. Front. Immunol..

[84] Fernandes M.R., Aggarwal P., Costa R.G.F., Cole A.M., Trinchieri G. (2022). Targeting the Gut Microbiota for Cancer Therapy. Nat. Rev. Cancer.

[85] Blake S.J., Wolf Y., Boursi B., Lynn D.J. (2023). Role of the Microbiota in Response to and Recovery from Cancer Therapy. Nat. Rev. Immunol..

[86] Ding R., Lian S.B., Tam Y.C., Oh C.C. (2024). The Cutaneous Microbiome in Skin Cancer—A Systematic Review. J. Dtsch. Dermatol. Ges..

[87] Aragon J., Weber A.M., Tipton M., Thomsen J., Ibrahim H., Weishaar K., Rao S., Suchodolski J.S., Stockman J., Ryan E.P. (2025). Impacts of Vincristine and Prednisolone Chemotherapy on the Canine Gut Microbiota in Dogs Undergoing Treatment for Lymphoma. Vet. Comp. Oncol..

[88] Isaiah A. (2018). *Clostridium hiranonis*, a Bile Acid 7α-Dehydroxylating Bacterium in Dogs. Ph.D. Thesis.

[89] Félix A.P., Souza C.M.M., de Oliveira S.G. (2022). Biomarkers of gastrointestinal functionality in dogs: A systematic review and meta-analysis. Anim. Feed Sci. Technol..

[90] Donnelly L., Couto J., Lemoine E., Erger C., Rindt H., Flesner B., McCleary-Wheeler A., Bryan J. (2021). Prospective Evaluation of the Fecal Microbiome in Dogs with Lymphoma Treated with CHOP Chemotherapy. Proceedings of the 2021 VCS Annual Conference.

[91] Leonov G., Starodubova A., Makhnach O., Goldshtein D., Salikhova D. (2026). Intratumoral microbiome: Impact on cancer progression and cellular immunotherapy. Cancers.

[92] Wang Y., Guo W., Wu X., Zhang Y., Mannion C., Brouchkov A., Man Y.-G., Chen T. (2019). Oncolytic bacteria and their potential role in bacterium-mediated tumour therapy: A conceptual analysis. J. Cancer.

[93] Rius-Rocabert S., Llinares Pinel F., Pozuelo M.J., García A., Nistal-Villan E. (2019). Oncolytic bacteria: Past, present and future. FEMS Microbiol. Lett..

[94] Jazowiecka J., Szala S. (2002). Oncolytic bacteria in cancer therapy. Contemp. Oncol..

[95] Withers S.S., Sparger E.E., Boudreaux B., Mason N.J. (2019). Utilizing Microbes to Treat Naturally Occurring Cancer in Veterinary Species. Curr. Clin. Microbiol. Rep..

[96] Khormi M.A., Al-Maaqar S.M., Al Johni A.R., Al-Tayyar N.A., Alhamad J.A., Ghyathuddin A.A., Alblawi Z., Behairy S.M., Alghamdi M.A., Alsubhi W.A. (2025). Oncolytic bacteria: A revolutionary approach to cancer therapy. Open Life Sci..

[97] Valencakova A., Dziakova A., Hatalová E. (2016). Oncolytic Activity of Bacteria used in Cancerous Disease Gene Therapy. Glob. J. Med. Res..

[98] Pierce K.M., Miklavcic W.R., Cook K.P., Hennen M.S., Bayles K.W., Hollingsworth M.A., Brooks A.E., Pullan J.E., Dailey K.M. (2021). The Evolution and Future of Targeted Cancer Therapy: From Nanoparticles, Oncolytic Viruses, and Oncolytic Bacteria to the Treatment of Solid Tumors. Nanomaterials.

[99] Łepeta K., Łasica A.M., Jagusztyn-Krynicka E.K. (2010). Zastosowanie produktów mikroorganizmów w terapiach antynowotworowych. Post. Mikrobiol..

[100] St Jean A.T., Zhang M., Forbes N.S. (2008). Bacterial Therapies: Completing the Cancer Treatment Toolbox. Curr. Opin. Biotechnol..

[101] Staedtke V., Sun N., Bai R. (2024). Hypoxia-Targeting Bacteria in Cancer Therapy. Semin. Cancer Biol..

[102] Runa F., Hamalian S., Meade K., Shisgal P., Gray P.C., Kelber J.A. (2017). Tumor Microenvironment Heterogeneity: Challenges and Opportunities. Curr. Mol. Biol. Rep..

[103] Zúñiga A., Camacho M., Chang H.-J., Fristot E., Mayonove P., Hani E.-H., Bonnet J. (2021). Engineered l-Lactate Responding Promoter System Operating in Glucose-Rich and Anoxic Environments. ACS Synth. Biol..

[104] Chien T., Harimoto T., Kepecs B., Gray K., Coker C., Hou N., Pu K., Azad T., Nolasco A., Pavlicova M. (2021). Enhancing the tropism of bacteria via genetically programmed biosensors. Nat. Biomed. Eng..

[105] Synchronized Lysis Circuit (SLC) Technology. https://live-biotherapeutic.creative-biolabs.com/synchronized-lysis-circuit-slc-technology.htm.

[106] Siguenza N., Brevi A., Zhang J.T., Pabani A., Bhushan A., Das M., Ding Y., Hasty J., Ghosh P., Zarrinpar A. (2024). Engineered bacterial therapeutics for detecting and treating CRC. Trends Cancer.

[107] Jarecka K., Romaniuk A., Paszel-Jaworska A., Rubiś B. (2018). Znaczenie autofagii i telomerazy w terapii nowotworów na przykładzie raka piersi. Postępy Biol. Komorki..

[108] Rosenberg S.A., Spiess P.J., Kleiner D.E. (2002). Antitumor Effects in Mice of the Intravenous Injection of Attenuated *Salmonella typhimurium*. J. Immunother..

[109] King I., Luo X., Feng M., Ittensohn M., Li Z., Belcourt M., Lin S., Le T., Pike J., Troy K. (2000). Tumour Therapy Using *Salmonella*. Exp. Opin. Emerg. Drugs.

[110] Saltzman D.A., Heise C.P., Hasz D.E., Zebede M., Kelly S.M., Curtiss R., Leonard A.S., Anderson P.M. (1996). Attenuated *Salmonella typhimurium* containing interleukin-2 decreases MC-38 hepatic metastases: A novel anti-tumor agent. Cancer Biother. Radiopharm..

[111] Saltzman D.A., Katsanis E., Heise C.P., Hasz D.E., Kelly S.M., Curtiss R., Leonard A.S., Anderson P.M. (1997). Patterns of hepatic and splenic colonization by an attenuated strain of *Salmonella typhimurium* containing the gene for human interleukin-2: A novel anti-tumor agent. Cancer Biother. Radiopharm..

[112] Li Y., Guo K., Chen H., Xie Y., Song C., Tang X., Ren D. (2001). Oral cytokine gene therapy against murine tumor using attenuated *Salmonella typhimurium*. Int. J. Cancer.

[113] Hoffman R.M. (2016). Tumor-Targeting *Salmonella typhimurium* A1-R: An Overview. Methods Mol. Biol..

[114] Tangney M., Gahan C. (2010). *Listeria monocytogenes* as a Vector for Anti-Cancer Therapies. Curr. Gene Ther..

[115] Van Pijkeren J.P., Morrissey D., Monk I.R., Cronin M., Rajendran S., O’Sullivan G.C., Gahan C.G.M., Tangney M. (2010). A Novel *Listeria monocytogenes*-Based DNA Delivery System for Cancer Gene Therapy. Hum. Gene Ther..

[116] Pillich H., Puri M., Chakraborty T. (2016). ActA of *Listeria monocytogenes* and Its Manifold Activities as an Important Listerial Virulence Factor. Curr. Top. Microbiol. Immunol..

[117] Stark F.C., Sad S., Krishnan L. (2009). Intracellular bacterial vectors that induce CD8^+^ T cells with similar cytolytic abilities but disparate memory phenotypes provide contrasting tumor protection. Cancer Res..

[118] Makino M., Kawai M., Kawamura I., Fujita M., Gejo F., Mitsuyama M. (2005). Involvement of reactive oxygen intermediate in the enhanced expression of virulence-associated genes of *Listeria monocytogenes* inside activated macrophages. Microbiol. Immunol..

[119] Kim S.H., Castro F., Paterson Y., Gravekamp C. (2009). High efficacy of a *Listeria*-based vaccine against metastatic breast cancer reveals a dual mode of action. Cancer Res..

[120] Janku F., Zhang H.H., Pezeshki A., Goel S., Murthy R., Wang-Gillam A., Shepard D.R., Helgason T., Masters T., Hong D.S. (2021). Intratumoral Injection of *Clostridium novyi*-NT Spores in Patients with Treatment-Refractory Advanced Solid Tumors. Clin. Cancer Res..

[121] Bettegowda C., Huang X., Lin J., Cheong I., Kohli M., Szabo S.A., Zhang X., Diaz L.A., Velculescu V.E., Parmigiani G. (2006). The genome and transcriptomes of the anti-tumor agent *Clostridium novyi*-NT. Nat. Biotechnol..

[122] Dailey K.M., Jacobson R.I., Johnson P.R., Woolery T.J., Kim J., Jansen R.J., Mallik S., Brooks A.E. (2021). Methods and Techniques to Facilitate the Development of *Clostridium novyi* NT as an Effective, Therapeutic Oncolytic Bacteria. Front. Microbiol..

[123] Roberts N.J., Zhang L., Janku F., Collins A., Bai R.-Y., Staedtke V., Rusk A.W., Tung D., Miller M., Roix J. (2014). Intratumoral injection of *Clostridium novyi*-NT spores induces antitumor responses. Sci. Transl. Med..

[124] Agrawal N., Bettegowda C., Cheong I., Geschwind J.-F., Drake C.G., Hipkiss E.L., Tatsumi M., Dang L.H., Diaz L.A., Pomper M. (2004). Bacteriolytic therapy can generate a potent immune response against experimental tumors. Proc. Natl. Acad. Sci. USA.

[125] Liu R., Cao Z., Wang L., Wang X., Lin S., Wu F., Pang Y., Liu J. (2022). Multimodal Oncolytic Bacteria by Coating with Tumor Cell Derived Nanoshells. Nano Today.

[126] Wei H., Chen L., Lian G., Yang J., Li F., Zou Y., Lu F., Yin Y. (2018). Antitumor Mechanisms of *Bifidobacteria* (Review). Oncol. Lett..

[127] Yazawa K., Fujimori M., Amano J., Kano Y., Taniguchi S. (2000). *Bifidobacterium longum* as a delivery system for cancer gene therapy: Selective localization and growth in hypoxic tumors. Cancer Gene Ther..

[128] Fu G.-F., Li X., Hou Y.-Y., Fan Y.-R., Liu W.-H., Xu G.-X. (2004). *Bifidobacterium longum* as an oral delivery system of endostatin for gene therapy on solid liver cancer. Cancer Gene Ther..

[129] Li X., Fu G.-F., Fan Y.-R., Liu W.-H., Liu X.-J., Wang J.-J., Xu G.-X. (2003). *Bifidobacterium adolescentis* as a delivery system of endostatin for cancer gene therapy: Selective inhibitor of angiogenesis and hypoxic tumor growth. Cancer Gene Ther..

[130] Shi Y., Zheng W., Yang K., Harris K.G., Ni K., Xue L., Lin W., Chang E.B., Weichselbaum R.R., Fu Y.-X. (2020). Intratumoral accumulation of gut microbiota facilitates CD47-based immunotherapy via STING signaling. J. Exp. Med..

[131] Shioya K., Matsumura T., Seki Y., Shimizu H., Nakamura T., Taniguchi S. (2021). Potentiated antitumor effects of APS001F/5-FC combined with anti-PD-1 antibody in a CT26 syngeneic mouse model. Biosci. Biotechnol. Biochem..

[132] Xiao S., Shi H., Zhang Y., Fan Y., Wang L., Xiang L., Liu Y., Zhao L., Fu S. (2022). Bacteria-driven hypoxia targeting delivery of chemotherapeutic drug proving outcome of breast cancer. J. Nanobiotechnol..

[133] Lu D., Wang L., Wang L., An L., Huo M., Xu H., Shi J. (2022). Probiotic Engineering and Targeted Sonoimmuno-Therapy Augmented by STING Agonist. Adv. Sci..

[134] Xie X., Guo J., Kong Y., Xie G.X., Li L., Lv N., Xiao X., Tang J., Wang X., Liu P. (2011). Targeted expression of *Escherichia coli* purine nucleoside phosphorylase and Fludara^®^ for prostate cancer therapy. J. Gene Med..

[135] Tai C.-K., Wang W., Lai Y.-H., Logg C.R., Parker W.B., Li Y.-F., Hong J.S., Sorscher E.J., Chen T.C., Kasahara N. (2010). Enhanced efficiency of prodrug activation therapy by tumor-selective replicating retrovirus vectors armed with the *Escherichia coli* purine nucleoside phosphorylase gene. Cancer Gene Ther..

[136] Swain A.L., Jaskólski M., Housset D., Rao J.K., Wlodawer A. (1993). Crystal structure of *Escherichia coli* L-asparaginase, an enzyme used in cancer therapy. Proc. Natl. Acad. Sci. USA.

[137] Thakur B.K., Malaise Y., Choudhury S.R., Neustaeter A., Turpin W., Streutker C., Copeland J., Wong E.O.Y., Navarre W.W., Guttman D.S. (2025). Dietary fibre counters the oncogenic potential of colibactin-producing *Escherichia coli* in colorectal cancer. Nat. Microbiol..

[138] Rakshit S., Ponnusamy M., Papanna S., Saha B., Ahmed A., Nandi D. (2011). Immunotherapeutic efficacy of *Mycobacterium indicus pranii* in eliciting anti-tumor T cell responses: Critical roles of IFNγ. Int. J. Cancer.

[139] Raskov H., Orhan A., Christensen J.P., Gögenur I. (2020). Cytotoxic CD8^+^ T cells in cancer and cancer immunotherapy. Br. J. Cancer.

[140] Kumar P., Tyagi R., Das G., Bhaskar S. (2014). *Mycobacterium indicus pranii* and *Mycobacterium bovis* BCG lead to differential macrophage activation in Toll-like receptor-dependent manner. Immunology.

[141] Zhou S., Gravekamp C., Bermudes D., Liu K. (2018). Tumour-targeting bacteria engineered to fight cancer. Nat. Rev. Cancer.

[142] He X., Wang J., Zhao F., Yu F., Chen D., Cai K., Yang C., Chen J., Dou J. (2012). Antitumor efficacy of viable tumor vaccine modified by heterogenetic ESAT-6 antigen and cytokine IL-21 in melanomatous mouse. Immunol. Res..

[143] Noguera-Ortega E., Guallar-Garrido S., Julián E. (2020). Mycobacteria-Based Vaccines as Immunotherapy for Non-urological Cancers. Cancers.

[144] De Leon J., Jiang G., Ma Y., Rubin E., Fortune S., Sun J. (2012). *Mycobacterium tuberculosis* ESAT-6 exhibits a unique membrane-interacting activity that is not found in its ortholog from non-pathogenic *Mycobacterium smegmatis*. J. Biol. Chem..

[145] Ushigusa T., Koyama Y., Ito T., Watanabe K., Chambers J.K., Hasegawa A., Uchida K., Kanegi R., Hatoya S., Inaba T. (2018). Innate immunity mediated by dendritic cells/macrophages plays a central role in the early period in tumor treatment using gene of *Mycobacterium tuberculosis* antigen. J. Vet. Med. Sci..

[146] Podder S., Rakshit S., Ponnusamy M., Nandi D. (2016). Efficacy of Bacteria in Cancer Immunotherapy: Special Emphasis on the Potential of Mycobacterial Species. Clin. Cancer Drugs.

[147] Josephs S.F., Ichim T.E., Prince S.M., Kesari S., Marincola F.M., Escobedo A.R., Jafri A. (2018). Unleashing endogenous TNF-alpha as a cancer immunotherapeutic. J. Transl. Med..

[148] Karbach J., Neumann A., Brand K., Wahle C., Siegel E., Maeurer M., Ritter E., Tsuji T., Gnjatic S., Old L.J. (2012). Phase I clinical trial of mixed bacterial vaccine (Coley’s toxins) in patients with NY-ESO-1 expressing cancers: Immunological effects and clinical activity. Clin. Cancer Res..

[149] Chen Y., Liu X., Guo Y., Wang J., Zhang D., Mei Y., Shi J., Tan W., Zheng J.H. (2021). Genetically engineered oncolytic bacteria as drug delivery systems for targeted cancer theranostics. Acta Biomater..

[150] Escobar-Díaz E., López-Martín E.M., Hernández Del Cerro M., Puig-Kroger A., Soto-Cerrato V., Montaner B., Giralt E., García-Marco J.A., Pérez-Tomás R., Garcia-Pardo A. (2005). AT514, a cyclic depsipeptide from *Serratia marcescens*, induces apoptosis of B-chronic lymphocytic leukemia cells: Interference with the Akt/NF-ΚB survival pathway. Leukemia.

[151] Wei X., Du M., Chen Z., Yuan Z. (2022). Recent Advances in Bacteria-Based Cancer Treatment. Cancers.

[152] Soto-Cerrato V., Montaner B., Martinell M., Vilaseca M., Giralt E., Pérez-Tomás R. (2005). Cell cycle arrest and proapoptotic effects of the anticancer cyclodepsipeptide serratamolide (AT514) are independent of p53 status in breast cancer cells. Biochem. Pharmacol..

[153] Richardson M.A., Ramirez T., Russell N.C., Moye L.A. (1999). Coley toxins immunotherapy: A retrospective review. Altern. Ther. Health Med..

[154] Liu Z., Wang L., Wu P., Yuan L. (2025). Precision Tumor Treatment Utilizing Bacteria: Principles and Future Perspectives. Appl. Microbiol. Biotechnol..

[155] Singh A.K., Awasthi R., Malviya R. (2023). Bioinspired microrobots: Opportunities and challenges in targeted cancer therapy. J. Control. Release.

[156] Park S.J., Park S.-H., Cho S., Kim D.-M., Lee Y., Ko S.Y., Hong Y., Choy H.E., Min J.-J., Park J.-O. (2013). New paradigm for tumor theranostic methodology using bacteria-based microrobot. Sci. Rep..

[157] Schmidt C.K., Medina-Sánchez M., Edmondson R.J., Schmidt O.G. (2020). Engineering microrobots for targeted cancer therapies from a medical perspective. Nat. Commun..

[158] Lin D., Feng X., Mai B., Li X., Wang F., Liu J., Liu X., Zhang K., Wang X. (2021). Bacterial-based cancer therapy: An emerging toolbox for targeted drug/gene delivery. Biomaterials.

[159] Qin X., Xu R., Wu J., Liu Y., Wang T., Tu H., Li J., Pang Z. (2025). Recent advances in engineering nano/microrobots for tumor treatment. Acta Pharm. Sin. B.

[160] Yin R., Jin H., Yu J., Shao L., Yu X. (2025). Progress in the application of the synergistic effects of nanomaterials and bacteria in tumor therapy. Cancer Nanotechnol..

[161] Wang Y., Xiao S., Yu W., Han B., Guo G. (2025). Engineering bacteria for enhanced tumor therapy: From surface modification to synthetic genetic circuits. J. Hematol. Oncol..

[162] Huang X., Pan J., Xu F., Shao B., Wang Y., Guo X., Zhou S. (2021). Bacteria-Based Cancer Immunotherapy. Adv. Sci..

[163] Zhou M., Tang Y., Xu W., Hao X., Li Y., Huang S., Xiang D., Wu J. (2023). Bacteria-based immunotherapy for cancer: A systematic review of preclinical studies. Front. Immunol..

[164] Wang B., Qin Y., Liu J., Zhang Z., Li W., Pu G., Yuanhe Z., Gui X., Chu M. (2023). Magnetotactic Bacteria-Based Drug-Loaded Micromotors for Highly Efficient Magnetic and Biological Double-Targeted Tumor Therapy. ACS Appl. Mater. Interfaces.

[165] Ma X., Liang X., Li Y., Feng Q., Cheng K., Ma N., Zhu F., Guo X., Yue Y., Liu G. (2023). Modular-designed engineered bacteria for precision tumor immunotherapy via spatiotemporal manipulation by magnetic field. Nat. Commun..

[166] Alphandéry E. (2020). Applications of magnetotactic bacteria and magnetosome for cancer treatment: A review emphasizing on practical and mechanistic aspects. Drug Discov. Today.

[167] Rajendran S., Sundararajan P., Awasthi A., Rajendran S. (2023). Nanorobotics in Medicine: A Systematic Review of Advances, Challenges, and Future Prospects. arXiv.

[168] Pal A., Sonowane R., Ramakrishna W. (2025). Targeting Cancers with Microrobots and Bacteriobots. Mol. Biotechnol..

[169] Liu Y., Yao J., Deng J., Zhou M., Shen K., Sun S., Gao X., Zhang Q., Xu H. (2026). Attenuated Bacteria-Based Tumor Therapy: Clinical Application Risks, Marketing Approval Restrictions, and Coping Strategies. Adv. Sci..

[170] Oh J.H., Cho J.-Y. (2023). Comparative Oncology: Overcoming Human Cancer through Companion Animal Studies. Exp. Mol. Med..

[171] Wang M., Song X., Liu X., Ma C., Ma J., Shi L. (2024). Engineered Oncolytic Bacteria for Malignant Solid Tumor Treatment. Interdiscip. Med..

[172] Roe J.M., Seely K., Bussard C.J., Martin E.E., Mouw E.G., Bayles K.W., Hollingsworth M.A., Brooks A.E., Dailey K.M. (2023). Hacking the Immune Response to Solid Tumors: Harnessing the Anti-Cancer Capacities of Oncolytic Bacteria. Pharmaceutics.

[173] Guo Y., Chen Y., Liu X., Min J.-J., Tan W., Zheng J.H. (2019). Targeted Cancer Immunotherapy with Genetically Engineered Oncolytic Salmonella Typhimurium. Cancer Lett..

[174] McKenzie B.A. (2025). Immunosenescence and Inflammaging in Dogs and Cats: A Narrative Review. J. Vet. Intern. Med..

[175] Hughes J.M.L. (2008). Anaesthesia for the geriatric dog and cat. Ir. Vet. J..

[176] Duong L., Radley H.G., Lee B., Dye D.E., Pixley F.J., Grounds M.D., Nelson D.J., Jackaman C. (2021). Macrophage function in the elderly and impact on injury repair and cancer. Immun. Ageing.

[177] Leonardi G.C., Accardi G., Monastero R., Nicoletti F., Libra M. (2018). Ageing: From Inflammation to Cancer. Immun. Ageing.

[178] Silverstein D. (2015). Systemic Inflammatory Response Syndrome & Sepsis. Today’s Vet. Pract..

[179] McGowan E. (2015). Systemic Inflammatory Response Syndrome & Sepsis. Today’s Vet. Pract..

[180] Jiang M., Yang Z., Dai J., Wu T., Jiao Z., Yu Y., Ning K., Chen W., Yang A. (2023). Intratumor Microbiome: Selective Colonization in the Tumor Microenvironment and a Vital Regulator of Tumor Biology. MedComm.

[181] Leong W.Y. (2025). Biohybrid micro-robots for targeted drug delivery in cancer therapy. Eng. Proc..

